# Exercise adherence Mobile app for Knee Osteoarthritis: protocol for the MappKO randomised controlled trial

**DOI:** 10.1186/s12891-022-05816-6

**Published:** 2022-09-20

**Authors:** Rana S. Hinman, Rachel K. Nelligan, Penny K. Campbell, Alexander J. Kimp, Bridget Graham, Mark Merolli, Fiona McManus, Karen E. Lamb, Kim L. Bennell

**Affiliations:** 1 Centre for Health, Exercise and Sports Medicine, Department of Physiotherapy, School of Health Sciences, Faculty of Medicine Dentistry & Health Sciences, The University of Melbourne, Melbourne, Australia; 2 Centre for Epidemiology and Biostatistics, Melbourne School of Population and Global Health, Biostatistics Unit, The University of Melbourne, Melbourne, Australia; 3 Methods and Implementation Support for Clinical Health Research Hub, Faculty of Medicine, Dentistry and Health Sciences, The University of Melbourne, Melbourne, Australia

**Keywords:** Osteoarthritis, Knee, Digital health, Rehabilitation, Physiotherapy, Clinical trial, Exercise, Adherence, Behaviour change, Telehealth, mHealth, Mobile app

## Abstract

**Background:**

In people with knee osteoarthritis (OA), ongoing exercise participation, particularly with strengthening exercises, is central to management. Patient adherence to prescribed exercise typically declines once consultations with a clinician have ceased. Mobile applications (apps) can incorporate behaviour change techniques that may assist adherence, potentially optimising clinical outcomes.

**Methods:**

This is a two-arm, pragmatic, superiority randomised trial. One hundred and eighty two Australians with chronic knee pain (clinical knee OA) and who have at least a mild level of physical dysfunction are being recruited. Participants are randomly allocated i) exercise (physiotherapist-prescribed exercise) or; ii) exercise plus app (physiotherapist-prescribed exercise plus access to the ‘My Exercise Messages’ mobile app). Exercise care comprises two videoconferencing consultations with a physiotherapist over two weeks (30 min each) for a strengthening exercise program, which is then conducted independently at home for 24 weeks without any further physiotherapist consultations. Participants are also provided with exercise resources to facilitate home-based exercise. Those randomised to exercise plus app will download the app after completing the two weeks of physiotherapy consultations and will be instructed by research staff to use the app for the 24 weeks of unsupervised home-based exercises. The app works by tracking completion of weekly exercise sessions, providing regular messages to facilitate weekly exercise and providing personalised messages to help overcome individual barriers to exercise participation. The two primary outcomes are i) self-reported physical function; and ii) number of days strengthening exercises were performed (previous fortnight), with a primary endpoint of 26 weeks and a secondary endpoint of 14 weeks. Secondary outcomes include knee pain severity; knee-related quality of life; global change; exercise program satisfaction; exercise self-efficacy; physical activity; sport and recreation function; another measure of exercise adherence; and willingness to undergo joint replacement. Process measures are also included.

**Discussion:**

Findings will determine if a theory-informed mobile app improves exercise adherence and physical function in people with knee OA who have received a home-based strengthening program.

**Trial Registration:**

Australian New Zealand Clinical Trials Registry, ACTRN12621000724875. Prospectively registered 9/06/2021.

## Background

Knee osteoarthritis (OA) is a major cause of pain and disability globally [1]. The pooled global prevalence of knee OA is 22.9% in people aged 40 and over, and in 2020, there were approximately 654 million people aged 40 years and older with knee OA [2]. People with knee OA frequently present to primary care clinicians for management of their condition [3]. Pain is a common complaint, particularly with movement and weight-bearing activities such as walking. Physical function is often impaired, which can adversely impact quality of life and an individual’s ability to participate in meaningful activities. Osteoarthritis is incurable and surgical arthroplasty is only advocated for people with end-stage OA in whom non-surgical treatments are not effective. Thus, clinical guidelines [4–7] for knee OA emphasise that self-management advice, exercise participation and weight control (if necessary) are essential management strategies.

Given the robust research attesting to the benefits of exercise on pain, physical function, performance and quality of life in people with knee OA [8–10], exercise is generally advised. Muscle strengthening is particularly important, given the lower limb weakness associated with knee OA [11–13] and the ability of muscle strengthening exercises to improve a range of important clinical outcomes [10]. Unfortunately, long-term adherence to exercise is poor [14, 15] in people with knee OA, particularly if support via contact with a health professional has ceased, is unavailable or is not possible. For example, a recent study in primary healthcare showed only 65% of people with knee OA were adherent to their physiotherapist-prescribed exercise program over 8 weeks [16] and other researchers have shown only 30% of people with hip or knee OA are adherent in the longer term [17].

People with OA encounter many barriers to exercise participation [18] including environmental factors, beliefs about exercise capability and consequences of exercise, and lack of motivation. Interventions to change behaviour are usually complex, which makes them challenging to implement in clinical settings and at scale [19]. Mobile applications (apps) are a popular tool for supporting people to make behavioural changes to manage chronic disease [20]. For example, mobile apps have been shown to be effective for improving mental and physical health [21], as well as nutritional behaviours and health outcomes [22]. Preliminary research suggests that mobile apps may be effective for promoting exercise adherence in gym users [23]. Importantly, mobile apps may be delivered at scale and low cost (or free) to the consumer. Most health and lifestyle apps aimed at changing health behaviours (such as physical activity, diet and sleep) contain few behaviour change techniques [24]. This may explain why there is currently no effective digital intervention for promoting exercise adherence in people with chronic musculoskeletal pain [25]. Furthermore, a recent systematic search of apps available on App Store and Google Play found that apps developed specifically for OA had the lowest quality and lowest potential for behaviour change compared to apps for other chronic conditions [26].

We recently developed a theory-informed mobile app called ‘My Exercise Messages’ that is available to consumers for free in the Apple App Store and Google Play. The app was adapted from our effective short message service (SMS) program that delivers personalised behaviour change messages to overcome barriers to exercise participation in people with knee OA [27]. Our research has shown that the SMS program of behaviour change messages improved adherence to unsupervised home exercise over 24 weeks in people with knee OA and obesity compared to people who received no SMS messages [28]. We have also shown that the SMS program, in combination with unsupervised web-based exercise, improved pain and function at 24-weeks in people with knee OA [29]. People with knee OA who used the SMS program found that the behaviour change messages to encourage exercise participation instilled a sense of support and accountability [30]. As scalable implementation of a behaviour change SMS program is challenging, we re-packaged the behaviour change messages into a mobile app, to permit rapid delivery at scale free of charge to the consumer. A mobile app also allowed inclusion of additional behaviour change techniques that were not amenable to SMS delivery, such as a graphical display for monitoring exercise adherence.

The primary aims of the current randomised controlled trial (RCT) are to determine if the ‘My Exercise Messages’ app confers any benefits after two consultations with a physiotherapist for prescription of a home strengthening program on:

i) physical function and/or;
ii) home exercise performance (exercise adherence) at 26 weeks.

Secondary aims are to:

i) determine if the app confers benefits on physical function and home exercise performance (exercise adherence) at 14 weeks;
ii) determine if the app confers any benefits on other clinical outcomes (knee pain severity; knee-related quality of life; global change; exercise program satisfaction; exercise self-efficacy; physical activity; sport and recreation function; another measure of exercise adherence; and willingness to undergo joint replacement) at 26 weeks;
iii) determine engagement with, and usefulness of, the app using a range of process measures; and
iv) explore potential moderators of the app’s effect on the clinical primary outcome (function).

## Methods

### Study design

A pragmatic superiority RCT is underway at University of Melbourne (sponsor). This protocol adheres to SPIRIT recommendations [31]. The trial was registered prospectively (Australian and New Zealand Clinical Trials Registry, ACTRN12621000724875). Consistent with OARSI recommendations for pragmatic RCTs evaluating exercise for OA [32], we are using broad selection criteria for participants, and physiotherapists from different geographical locations, to maximise external validity. Any protocol amendments will be described in our internal trial protocol document, notified to the institutional ethics committee and updated in the trial registry.

### Participants

One hundred and eighty two people with chronic knee pain are currently being recruited from the Australian community via community advertisements, clinician networks and our research volunteer database. Initial screening of volunteers occurs via an online form, then via telephone. Inclusion criteria are adapted from our other ongoing RCT [33] and are:

i) fulfill clinical classification criteria for knee OA [5];
  i. aged at least 45;
  ii. experience activity-related knee pain; and
  iii. experience morning knee stiffness lasting no longer than 30 min.
ii) knee pain history ≥ 3 months;
iii) knee pain experienced most days (of the prior month);
iv) at least mild physical dysfunction (score > 20 out of 68 on Western Ontario McMaster Universities (WOMAC) function subscale);
v) able to access a computer/laptop/tablet/smartphone with internet connection for videoconferencing with the physiotherapist;
vi) have a smartphone with software compatible for downloading/using the app if allocated to the intervention group;
vii) willing to participate in videoconferencing for physiotherapy appointments; and
viii) willing to download and engage with an app if allocated to the intervention group.
  Exclusion criteria are:

- i)  inability to understand English;
- ii)  planned/waiting list for knee/hip surgery in the next 6 months;
- iii)  have undergone a joint replacement on the affected knee at any time;
- iv)  recently undergone knee surgery (past 6 months);
- v)  are seeing a physiotherapist or performing knee strength exercises (currently or in prior 6 months);
- vi)  self-report a history of inflammatory arthritis (e.g. rheumatoid arthritis);
- vii)  self-report a neurological condition that affects the legs;
- viii) self-report any unstable/uncontrolled cardiovascular problems;

ix) self-report a fall (past 12 months) and do not obtain permission from their doctor to participate;
x) are house-bound due to immobility and do not obtain permission from their doctor to participate; and/or
xi) pre-exercise screening reveals a medical condition that may pose an exercise risk [34] and the volunteer does not obtain permission from their doctor to participate.

Anyone who has fallen (past 12 months), is house-bound because of immobility or who fails pre-exercise screening is asked to visit their doctor for medical permission to participate (and return a clearance letter signed by the doctor to the research team). If a volunteer reports bilateral knee problems, the most symptomatic eligible knee will be evaluated for the outcome measures.

### Summary of procedures

**Fig. 1:**
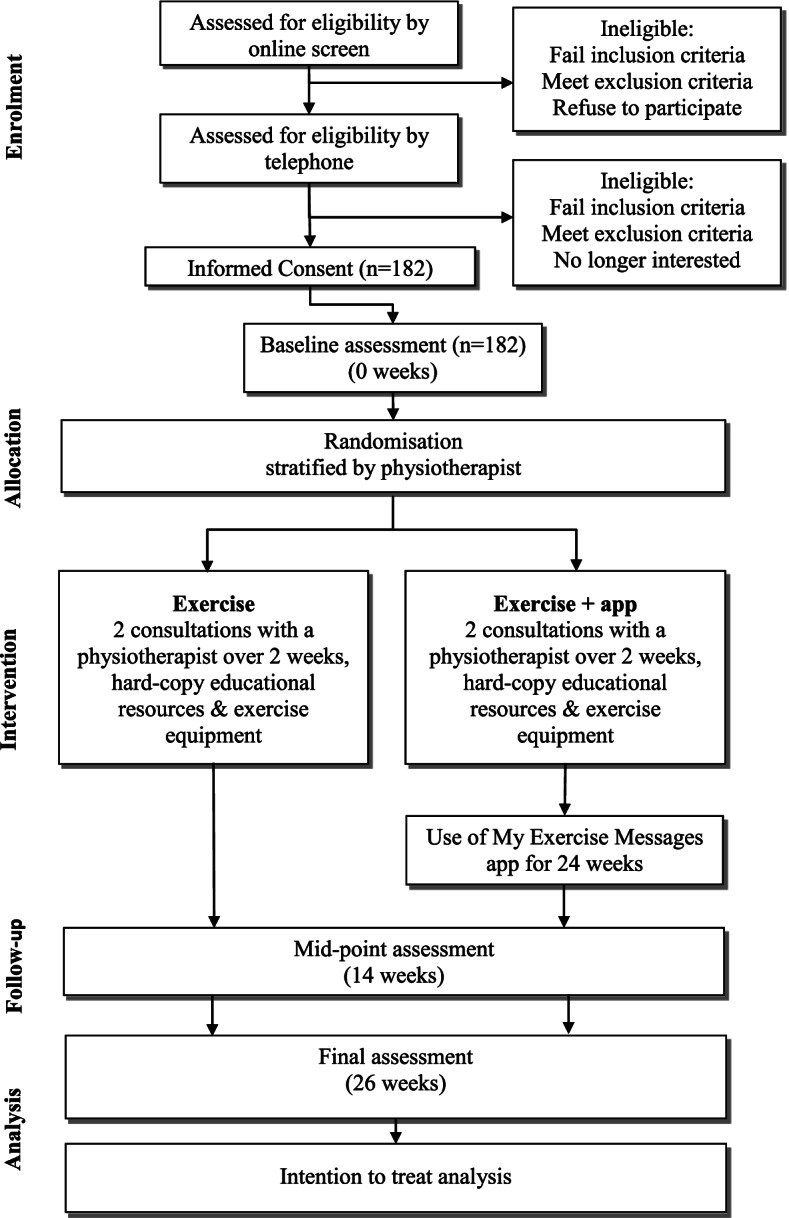
Flow of participants through the trial

Figure 1 provides an overview of the RCT. All potential participants are provided with verbal and written information about the trial from research staff. After passing telephone screening (with a verbal description of the trial), participants are provided a lay trial summary and consent form (post or email). Approval for the project was provided by the University of Melbourne Human Research Ethics Committee (HREC Project ID 20727.2). All participants provide written informed consent. Participants are not restricted from using co-interventions throughout the trial and use of these will be measured (described below).

### Randomisation, blinding and allocation concealment

Participants are enrolled in the trial once they have given informed consent and completed the baseline questionnaire. The randomisation schedule (allocation in a 1:1 ratio to either i) exercise or ii) exercise plus app) was prepared by an independent biostatistician (permuted random block sizes), stratified by physiotherapist. The randomization schedule is stored securely online with password-protection (REDCap™) and maintained by a researcher who does not recruit participants or administer outcome measures. The same researcher reveals group allocation after baseline assessment. At this point, allocation is revealed only to this researcher. Another researcher reveals allocation to those randomised to the exercise plus app group approximately two weeks later, after the participant has completed their physiotherapy consultations (to ensure physiotherapist blinding, see below).

As this is a pragmatic trial, participants are not blinded to group allocation. However, limited disclosure is used to reduce the risk of participants allocated to the exercise only group from searching for and downloading the ‘My Exercise Messages’ app. Participants in the trial are told the trial is assessing an “exercise app” but they are not told the name of the specific app under investigation nor its specific functions or purpose. As the primary and secondary outcomes are self-reported, and participants are not blinded, by default the assessors of these outcomes are not blinded. The physiotherapists who provide the exercise programs in both trial arms are blinded to group allocation. Participants in the exercise plus app group are not told of their group allocation until after they have completed their two physiotherapy sessions, to ensure they do not disclose their group allocation to their physiotherapist. The statistical analysis plan will be written and published while the biostatisticians are blinded. Main statistical analyses will be undertaken blinded to group name.

### Exercise group

Exercise-based care is delivered by one of 10 registered musculoskeletal physiotherapists from primary care settings in Victoria and Queensland, who prescribe a strengthening exercise program via videoconferencing to individual participants. Two consultations (approx. 10–14 days apart) with the physiotherapist occur using Zoom videoconferencing software (Zoom Video Communications, Inc., USA), each of approximately 30 min duration. The physiotherapists are a sub-set of physiotherapists delivering care in another of our RCTs [33] and are trained [35] in the delivery of evidence-based best-practice management of knee OA (education, strengthening exercises, physical activity) via videoconferencing. Participants are randomly assigned a physiotherapist and it is intended that the same therapist undertakes both consultations with any given participant, except where there is an unanticipated absence of the physiotherapist (whereby the consultation is then scheduled with another randomly assigned trial physiotherapist). Participants are provided a pre-consultation survey to complete and return prior to their first consultation. The treating physiotherapist uses this information as a basis for initial assessment. Each participant is provided resources to facilitate home-based strengthening exercises, including a booklet describing how to set up and use Zoom software; a booklet containing exercises and information about how to safely perform and modify exercises and four elastic bands for home exercises.

Table 1 describes the main components of each physiotherapy consultation, which are based on our previous and ongoing research [33, 36, 37] and were informed by behaviour change theory [38]. Physiotherapists prescribe a home-based program of 5–6 strengthening exercises [33] from a trial-specific exercise booklet (that contains individual exercises based upon our prior clinical trials [39–41]) to be performed three times/week. Participants are asked to perform all of their prescribed exercises each time they exercise. The program includes exercises targeting the quadriceps, hip/gluteal and calf muscles. Physiotherapists aim for the intensity of each exercise to be 5–7 out of ten (hard to very hard) [42] and individualise dosage of each exercise. Progression of the program and dosage at the second consultation follows recommended guidelines [43]. Physiotherapists can change the exercises within the program at the second consultation, and participants are advised how they can progress their program independently after consultations have finished. Participants are advised to continue their exercise program (three times/week) until the 26-week final assessment. No further support is provided by the physiotherapist after conclusion of the second consultation.

**Table 1:** Main components of each videoconferencing consultation with the physiotherapist, adapted from [33]

	**FIRST consultation (30 min)**	**SECOND Consultation (30 min)- approximately 10–14 days later**
Assessment	Introduction and outline aim of sessions is strengthening exercises Review pre-consult survey- choose a question for reassessment at second consult Obtain subjective information as relevant Functional observation: walking, squatting, sit to stand, single leg standing balance, anything else as relevant	Review… - knee pain - progress with strengthening program - any problems with any particular exercises? - too easy? Too hard? Reassess pain and a functional question from the pre-consult survey Re-assess sit to stand and any other functional tasks as relevant
Strengthening exercises	Prescribe a program of 5–6 exercises from the participant exercise booklet: -2 quadriceps -1 hip/gluteal -1 hamstring/gluteal -1 calf -1 optional other from the booklet Individualise the dosage of each exercise in terms of sets/repetitions, noting the whole program must be performed three/week. Prescribe exercise band colour for each exercise (can be different colour for different exercises) Watch participant perform a set of each exercise and ensure it is at a hard to very hard level Discuss how to manage pain/flare-ups with exercise	Review exercise program and modify/progress/change exercises as needed Watch patient perform a set of entire exercise program and ensure it is at a hard to very hard level Discuss with the participant how to modify & progress the exercise program over the next 24 weeks, noting the whole program must be performed three/week Advise participant to continue exercise program for next 24 weeks

Consultations occur via Zoom videoconferencing software. Physiotherapists are provided with an online video library of exercises so they can visually demonstrate the exercises to participants using screen sharing. Physiotherapists complete consultation notes after each consultation. Treatment protocol fidelity will be measured via the number and proportion of i) strength programs prescribed by the second consultation; ii) consultations where the intensity of each exercise was between 5–7 out of ten. The mean (standard deviation, SD) exertion rating for each person’s exercise program will be calculated across consultations.

### Exercise plus app group

In addition to physiotherapist-delivered exercise care described above, participants in the exercise plus app group are instructed by research staff to download the ‘My Exercise Messages’ app (from the Apple App Store or Google Play depending on their device), within 1–2 days of completing their second physiotherapy consultation. Participants are strongly encouraged to download the app onto their smart phone rather than a tablet. Over the phone, research staff explain how to use the app and participants are instructed to commence use of the app immediately and to use it for the maximum 24-week program of exercise tracking and behaviour change messages available within the app.

The ‘My Exercise Messages’ app is free to download from the Apple App Store (Apple devices) and Google Play (Android devices). The app was created by the research team to help people with hip and/or knee OA adhere to their weekly exercise goals, including exercise prescribed by health professionals. The app works by providing a function to record completion of weekly exercise sessions, providing regular messages to facilitate weekly exercise participation and providing personalised messages to help overcome any barriers to exercise participation encountered by the user. Users tell the app how many times each week they aim to exercise and how long they wish to use the program for (up to 24 weeks). The ‘My Exercise Messages’ app was adapted from our SMS program (which was developed according to behaviour change theory and explained in detail in a previous publication [27]) that is proven to enhance exercise adherence [28]. A summary of the key features of the app and how they map to behaviour change techniques can be found in Table 2. The ‘My Exercise Messages’ app scores 17 out of 21 (where higher scores indicate greater potential for promoting behaviour change) on the App Behaviour Change Scale [44] (Table 3). This compares to a mean of 4.22 for other mobile apps designed for people with OA [26] and a mean of 7.8 for health and well-being apps designed to modify lifestyle behaviours, including physical activity [45].

**Table 2:** Features of the ‘My Exercise Messages’ app mapped to behaviour change techniques [46]

Feature within the app	Behaviour change technique
Input a weekly exercise goal (target)	Goal setting (behaviour)
Messages/function to record weekly exercise sessions	Prompts/cues Self-monitoring of behaviour
Graph displaying weekly exercise sessions, relative to the exercise goal (target)	Review of behavioural goals Feedback on behaviour Discrepancy between current behavior and goal
Motivational/praise/encouragement messages when the exercise goal (target) is achieved	Social reward
Message acknowledging partial achievement of exercise goal (target) where appropriate	Social reward
Selection and input of main exercise barrier when exercise goal (target) not achieved	Problem solving
Tailored messages with tips to overcome reported exercise barrier^†^	Goal setting (behaviour) Problem solving Goal setting (outcome) Action planning Self-reward Restructuring the physical environment Distraction Verbal persuasion about capability Focus on past success Self-talk Feedback on behaviour Self-monitoring of outcome(s) of behaviour Social support (unspecified) Instruction on how to perform behaviour Behavioural experiments Information on health consequences Prompts/cues Habit formation Graded tasks
Generic regular messages to facilitate ongoing exercise adherence	Information on health consequences Self-reward Instruction on how to perform behaviour Social comparison
Reducing frequency of messages as the program progresses	Reduce prompts/cues
Benefits of exercise and physical activity for osteoarthritis information section	Information on health consequences Instruction on how to perform behaviour

^†^Note- users do not necessarily receive messages containing all behaviour change techniques, as messages sent are dependent on the barriers reported by users

**Table 3:** App behaviour change scale score [44] for the ‘My Exercise Messages’ app

Question	Response 1 = yes; 0 = no	Comment	Example behaviour change message (where relevant)
**1. Knowledge and information**			
1.1 Does the app have the ability to customise and personalise some features?	1	Customisable features: - person’s name to personalise messages - number of weekly exercise sessions (i.e. set a goal of between 1–7 sessions/week)* - length of program (between 1–24 weeks)* - selection of an exercise barrier (if low exercise adherence reported) from a defined list triggers a tailored exercise adherence support message (BCT)	
1.2 Was the app created with expertise and/or Does the app provide information that is consistent with national guidelines?	1	Created by researchers at University of Melbourne with expertise in osteoarthritis, exercise and BCTs Based on clinical practice guidelines that recommend physical activity and exercise for all people with hip/knee OA	
1.3 Does the app ask for baseline information?	0		
1.4 Does the app provide instruction on how to perform the behaviour?	1	Instruction on how to perform exercise is provided in: - Facilitator messages × 3 - Barrier response messages × 7^†^ Instructional tips provided in ‘Benefits of exercise’ information page under the settings tab	Facilitator message: It’s up to you to stay on track with your exercises with a little bit of help from us! People find it useful to make the exercises a priority and do them before starting the busy day. Try doing them first thing in the morning this week and see if it helps you stay on track ‘The exercises aren’t helping’ barrier response message: It sounds like something needs to change. Your exercises may not be challenging you enough to see improvements. This next week step it up, increase the weights or number of repetitions of an exercise
1.5 Does the app provide information about the consequences of continuing and/or discontinuing behaviour?	1	Information about health consequences is targeted in: - Facilitator messages × 13 - Barrier response messages × 28^†^ Information about exercise consequences (including risks and benefits) provided in ‘Benefits of exercise’ information page under the settings tab	Facilitator message: (name), let’s bust this myth—surgery is not inevitable if you have osteoarthritis! Exercise is one of the most effective ways to reduce your joint pain and prevent surgery ‘My pain limited my ability to do my exercises’ barrier response message: It sounds like your joint’s a bit sore already. Remember with osteoarthritis some days are worse than others. Consider doing a bit of exercise on the days when the pain isn’t as severe. Doing some exercise now could help there be less bad days in the long run
**2. Goals and planning**			
2.1 Does the app ask for willingness for behaviour change?	0		
2.2 Does the app allow for the setting of goals?	1	Setting of weekly exercise goal (between 1–7 exercise sessions/week)	
2.3 Does the app have the ability to review goals, update, and change when necessary?	1	Ability to change weekly exercise goal under the profile tab at any stage	
**3. Feedback and monitoring**			
3.1 Does the app give the user the ability to quickly and easily understand the difference between current action and future goals?	1	Via the graph of weekly exercise days plotted relative to the exercise goal	
3.2 Does the app have the ability to allow the user to easily self-monitor behaviour?	1	Via the graph of weekly exercise days plotted relative to the exercise goal	
3.3 Does the app have the ability to share behaviours with others (including social media or forums) and/or allow for social comparison?	0		
3.4 Does the app have the ability to give the user feedback—either from a person or automatically?	1	Automatic feedback via personalised messages in response to the user inputting data about - the weekly number of exercise sessions achieved - the main exercise barrier encountered (if the exercise goal was not achieved)	
3.5 Does the app have the ability to export data from app?	0		
3.6 Does the app provide a material or social reward or incentive?	1	Positive reinforcement/congratulations are provided when the weekly exercise goal is reached or exceeded	
3.7 Does the app provide general encouragement?	1	Visual encouragement provided via - the graph where weekly exercise days are plotted relative to the weekly exercise goal - the home tab where progress each week is logged against a visual target (the weekly exercise goal) Text-based encouragement is provided within all exercise facilitator messages (user receives 2/week during weeks 1–4; 1/week during weeks 5–16; 1/fortnight during weeks 17–24)	
**4. Actions**			
4.1 Does the app have reminders and/or prompts or cues for activity?	1	Weekly reminders provided to log exercise sessions Regular facilitator messages prompt exercise behaviour (user receives 2/week during weeks 1–4; 1/week during weeks 5–16; 1/fortnight during weeks 17–24)	
4.2 Does the app encourage positive habit formation?	1	Habit formation BCT encouraged in week 8 and week 24 facilitator messages Habit formation targeted in two exercise barrier response messages^†^	Facilitator message: Research shows that people who integrate exercise into their daily lives find it easier to exercise long term. Research also shows people see greater improvements the longer they exercise. The aim is to try make exercise a life habit ‘I forgot to do my exercises’ barrier response message: It can be hard to remember. We suggest making the exercises a habit. Set aside the same time each day to do them. It’s much harder to forget when something is a daily routine
4.3 Does the app allow or encourage for practice or rehearsal, in addition to daily activities?	1	Ability to log more exercise sessions than the goal each week (up to 7 sessions over 7 days)	
4.4 Does the app provide opportunity to plan for barriers?	1	Barriers addressed if weekly exercise goal not met by:—user being asked to reflect on and select their major barrier to exercise for that week - based on the barrier selected, a targeted BCT message is then sent with suggestions for overcoming the specific barrier	
4.5 Does the app assist with or suggest restructuring the physical or social environment?	1	Environmental restructuring is adressed in 10 barrier response messages (in response to the user selecting the barrier ‘forgot’) ^†^	‘I forgot to do my exercises’ barrier response message: It can be hard to remember to exercise. Try putting your exercise equipment somewhere you will see it every day, this can help trigger your memory to do your exercises
4.6 Does the app assist with distraction or avoidance?	1	Distraction BCT targeted in five barrier response messages (in response to the user selecting the barrier ‘boring’ or ‘pain limits’)^†^	‘I found the exercises boring’ barrier response message: Doing the same exercise again and again can be a drag. Try distracting yourself. What about doing your exercises while watching your favourite TV show?
**Total score (out of 21)**	**17**		

*BCT* behaviour change technique

^†^Note- users do not necessarily receive all barrier response messages containing BCTs. Barrier response messages are only sent in response to specific barriers selected by users reporting < 3 exercise sessions in a week (low adherence)

^*^Note in this RCT, participants are instructed to set a weekly exercise session goal of 3 sessions and to set the length of the program for 24 weeks

For this trial, participants are instructed to input an exercise goal of three sessions per week and set the program for 24 weeks. Each week, participants receive a notification prompting them to enter the app and record how many exercise sessions they completed in the prior week. Participants also have the option of logging their exercise sessions in real-time. A graph tab displays exercise sessions completed each week so participants can monitor their progress. If participants have not achieved the goal of three sessions/week, the app asks them (weekly up to week 9 and fortnightly thereafter) to select their major exercise barrier from a predefined list (based on our research into the major barriers to exercise in OA [18]) and then sends behaviour change messages tailored to the selected barrier to help them adhere to their exercise program. Irrespective of goal attainment, the app sends extra messages throughout the week to remind and facilitate users to achieve their exercise target.

### Outcome measures

Table 4 lists the outcome measures in this RCT, relative to the timing of enrolment and interventions. The primary and secondary outcomes are collected on a web-based platform (REDCap™) or via post (if participants prefer paper versions of the questionnaires). The primary end-point is 26 weeks post-randomisation (for primary and secondary outcomes) and the secondary end-point is 14 weeks (for primary outcomes only). To encourage participant retention in the trial and completion of outcome measures, participants who complete the electronic survey at 26 weeks will be provided with a $AUD50 gift voucher to compensate them for the time they have invested in the trial.

**Table 4:**
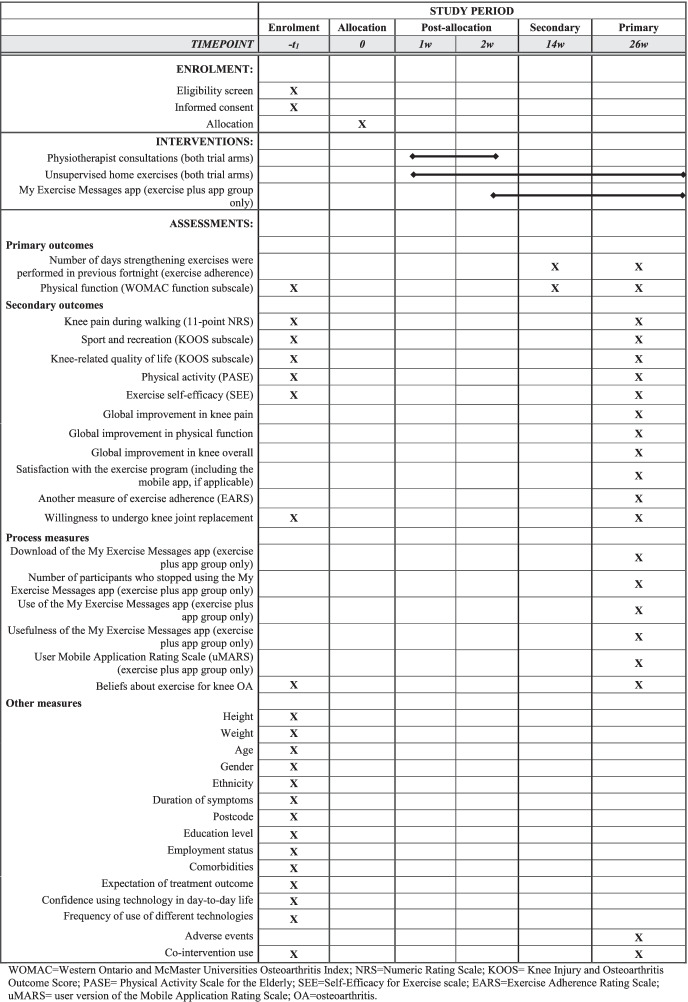
Schedule of enrolment, interventions and assessments

### Primary outcomes

There are two primary outcomes for this RCT:

i) *Number of days strengthening exercises were performed in previous fortnight (exercise adherence)*
  At 14 and 26 weeks, participants are asked “In the past two weeks, how many days did you perform the strengthening exercise program your physiotherapist prescribed for your knee problem?” Options range from “0” to “6 or more”. To aid interpretation of descriptive data, responses will also be converted into a percentage of the prescribed six sessions, where 100% = exercises completed on 6 or more days of the past fortnight.
ii) *Physical function over the past week*

Physical dysfunction is measured via the Western Ontario and McMaster Universities (WOMAC) Osteoarthritis Index (Likert version 3.1) [47]. It is a valid, reliable and responsive self-reported OA-specific tool [48]. The physical function subscale has 17 questions which evaluate knee function over the prior week during a variety of daily activities (response options ranging from ‘no dysfunction’ to ‘extreme dysfunction’). Total score ranges from 0 to 68 and higher scores indicate poorer function. Change scores will be determined with data from baseline, 14 weeks and 26 weeks.

### Secondary outcomes

A range of secondary outcomes are being collected at baseline and 26 weeks (unless otherwise indicated). For continuous secondary outcome measures (excluding the Exercise Adherence Rating Scale (EARS), see below), change scores will be calculated using data from baseline and 26 weeks.

i) *Knee pain during walking*
  Average pain on walking (over the prior week) is measured with an 11-point numerical rating scale that has terminal descriptors of ‘no pain’ (score = 0) and ‘worst pain possible’ (score = 10).
ii) *Sport and recreation function*
  The Knee Injury and Osteoarthritis Outcome Score (KOOS) [49] sport and recreational activities subscale measures function in sports and recreational activities. It comprises five questions ascertaining function with sport and recreational activities over the last week, with 5-point Likert response options ranging from ‘none’ to ‘extreme’. Total score ranges from 0 to 100, with lower scores indicating more difficulty with sports and recreational activities.
iii) *Knee-related quality of life*
  The KOOS [49] quality of life subscale measures knee-related quality of life. It asks four questions about knee-related quality of life over the previous week, with 5-point Likert response options. Total score ranges from 0 to 100, with lower scores indicating poorer quality of life.
iv) *Physical activity*
  Physical activity over the prior week is measured via the Physical Activity Scale for the Elderly (PASE) [50]. It comprises 10 questions about frequency and duration of recreational, household and occupational physical activity over the prior 7 days. Total scores range from 0 to 793 and higher scores are indicative of more physical activity.
v) *Exercise self-efficacy*
  The Self-Efficacy for Exercise Scale [51] measures self-efficacy expectations about ability to continue exercising in the face of perceived barriers. This 9-item tool has scores which range from 0 to 90, with higher scores indicating better exercise self-efficacy.
vi) *Participant-perceived global changes*
  At 26 weeks, participants rate their global change from baseline in i) knee pain, ii) physical function, and iii) their knee overall, via individual 7-point Likert scales (ranging from “much worse/less” to “much better/more”). On each scale, participants who are moderately better or much better will be classed as improved and the rest as not improved.
vii) *Satisfaction with exercise program (including the mobile app, if applicable)*
  At 26 weeks, satisfaction with the entire exercise program (including the mobile app for those who were randomised to receive it) is rated with a 7-point Likert scale (ranging from “extremely unsatisfied” to “extremely satisfied”). Participants who are moderately satisfied or extremely satisfied will be classed as satisfied and the rest as not satisfied.
viii) *Exercise Adherence Rating Scale (EARS)*
  A secondary measure of exercise adherence is included (in addition to the primary adherence measure). At 26 weeks, participants rate their adherence to their prescribed home strengthening exercises using Section B of the EARS [52]. This has six items and each is scored with a 5-point scale (terminal descriptors of ‘strongly agree’ to ‘strongly disagree’). The total score ranges from 0 to 24, with higher scores indicating better adherence.
ix) *Willingness to undergo knee joint replacement*

At baseline and 26 weeks, participants will answer the question “How willing are you to have a knee joint replacement on your knee in the near future?” using a 5-point Likert scale (ranging from “definitely not willing” to “definitely willing”). Participants will be dichotomised into those not willing (definitely not willing; probably not willing) or willing/unsure (unsure; probably willing; definitely willing).

### Other measures

Other outcomes that will be measured for descriptive purposes, include:

i) *Sample characteristics*
  Variables measured at baseline include; height; weight; age; gender; ethnicity; symptom duration; residential postcode; education level; employment status; comorbidities (Self-Administered Comorbidity Questionnaire [53]); treatment outcome expectations (5-point ordinal scale from “no effect at all” to “complete recovery”); confidence using technology in daily life (rated on a 4-point Likert scale with options of not at all confident, somewhat confident, moderately confident and extremely confident) and use of technologies for health (over the previous month) rated as yes or no for each of i) searching the internet for health information; ii) using an app to manage health; iii) social networking (eg Facebook, Twitter) for health information/support; iv) wearable devices/ trackers for managing health; v) following/watching YouTube for health information and/or; vi) subscription to online health management programs.
ii) *Co-interventions*
  At baseline and 26 weeks, participants will complete a custom table to indicate how often over the prior month they used different pain and arthritis medications and other co-interventions (including use of healthcare apps) for their knee pain. Participants who used a drug/supplement at least once a week will be classed as a current user of the relevant medication. Participants who used any other co-intervention once in the past month will be reported as a recent user.
iii) *Adverse events*
  Adverse events are considered to be “any problem experienced in the study knee or elsewhere in the body deemed by the participant to be a result of participating in the trial AND at least one of i) that caused increased pain and/or disability for two days or more, and/or ii) resulted in the participant seeking treatment from a health professional” [33]. Adverse events will be established using survey questions to participants at 26 weeks. As the interventions in this trial are low-risk, serious adverse events (incapacitating, life-threatening, hospitalisation or death) are extremely unlikely and a Data & Safety Monitoring Committee is not required.
iv) *Process measures- exercise plus app group only*
  A range of self-reported process measures will be collected from the exercise plus app group only at 26 weeks. These include: i) downloads of the ‘My Exercise Messages’ app; ii) number of people who stop using the app (and reasons why); iii) number of days the app was used in the prior 14 days (via monthly survey until 26 weeks); iv) usefulness of the app; and v) user engagement, functionality, information quality and perceived impact of the app using the user Mobile Application Rating Scale [54].
v) *Process measure- both groups*

At baseline and 26 weeks, all participants will answer the question “How effective do you believe exercise is for managing knee osteoarthritis?” using a 4-point Likert scale (with response options of “not at all effective”; “somewhat effective”; “moderately effective”; and “highly effective”).

### Sample size calculations

A sample of 182 (91 per arm) is necessary to detect a minimum important difference of 1.2 sessions in the adherence primary outcome (number of days strengthening exercises performed in previous fortnight) at 26 weeks between groups (based on data from our trial evaluating SMS behaviour change messages [27]) with 80% power and an alpha of 0.025 (alpha of 0.05 split between the two primary outcomes), assuming a between-participant SD of 2.4 [27] and 15% attrition [28]. This sample size ensures more than 80% power to detect the minimal important difference on the clinical primary outcome of 6 units in change (follow-up minus baseline) in physical function on WOMAC [55] between the two arms, assuming a between-participant SD of 12 units [28], a baseline to 26-week correlation of 0.5 [28], an alpha of 0.025 and 15% attrition [28]. Physiotherapists treat participants in both groups, thus we have not modified the sample size for physiotherapist clustering.

### Statistical analysis plan

A biostatistician will analyse data while blind to group details. A Statistical Analysis Plan will be written and published on our research centre’s website prior to data analysis commencing and while blind to group allocation. Main between group comparative analyses will be based on intention-to-treat. Multiple imputation will occur if a primary outcome has more than 5% of data missing at 26 weeks. For the physical function primary outcome, differences in mean change (follow-up minus baseline) in function will be compared across groups via a mixed-effects linear regression model, adjusted for baseline values and the stratifying variable (physiotherapist). Terms for time and group by time interaction will be included as fixed effects, with random effects for participants and physiotherapists. A similar mixed-effects linear regression model will be used for the adherence primary outcome, except, as there is no baseline score, there will be no adjustment for baseline values and the outcome will be follow-up score. For secondary continuous outcomes, differences in mean change (follow-up minus baseline) will be compared between-groups using linear regression modelling adjusted for the outcome at baseline, and the stratifying variable. As there is no baseline score for the secondary continuous outcome EARS, the outcome measure is EARS score at 26-weeks and there will be no adjustment for baseline values. The proportion of participants in each group that show an improvement that reaches or exceeds the minimum clinically important difference in average walking pain (≥ 1.8 NRS units [56]) and physical function (≥ 6 WOMAC units [55]) will be calculated. For these and other binary outcomes, groups will be compared using risk differences and risk ratios, calculated from logistic regression models adjusted for the outcome at baseline (where available) and the stratifying variable of physiotherapist and fitted using generalised estimating equations.

We will explore potential treatment effect moderators on change in the physical function primary outcome at 26 weeks, based on the following a priori hypotheses:

- a. Baseline beliefs about exercise

Hypothesis- Participants who think that exercise is less effective for managing knee OA at baseline will report less improvement in physical function with the exercise plus app intervention, compared to those believing exercise is more effective (relative to the exercise group).

- b. Baseline confidence using technology

Hypothesis- Participants who are less confident using technology at baseline will report less improvement in physical function with the exercise plus app intervention, compared to participants with greater confidence (relative to the exercise group).

Interaction terms between randomised group and these variables will be included in linear regression models for the physical function primary outcome at 26 weeks, for each potential effect modifier separately.

### Patient and public involvement

We iteratively engaged end-users during the development of the ‘My Exercise Messages’ mobile app. Initially, when we developed the library of behaviour change messages, we consulted a total of 12 people (7 academics with OA expertise, four clinical physiotherapists, and one person with knee OA) individually about message wording [27]. Samples of messages were also reviewed by a behaviour change expert to ensure accuracy. When developing the mobile app, we sought feedback on the initial app design and functionality from four clinical physiotherapists and nine academics (mostly physiotherapists). After modifications and inclusion of additional features, we then pilot tested a revised prototype of the app with five people with OA. After each stage of review, we considered the feedback received and incorporated changes into the subsequent iteration (whether it was the wording of the messages, or presentation/usability/information within the mobile app itself) to ensure that the final product was both feasible to use and acceptable to end-users.

### Timelines

Ethical approval was gained in January 2021. Participant recruitment began in August 2021 and is due for completion in 2023. The trial should be completed in 2024.

### Dissemination

Findings will be shared via publications in peer-reviewed journals and presentations at conferences. We will follow International Committee of Medical Journal Editors recommendations for authorship. Participants will be provided with a plain language statement of results. The mobile app is available in Apple App Store and Google Play.

## Discussion

This paper has described the rationale and protocol for an Australian two-arm pragmatic superiority RCT evaluating if a theory-informed mobile app (‘My Exercise Messages’) confers additional benefits to a physiotherapist-prescribed home-based strengthening exercise program at 26 weeks. Exercise, particularly strengthening, is advocated as core management for all people with knee OA [4–7], yet adherence to exercise often declines once contact with a health professional has ceased. Mobile apps can incorporate positive behaviour change techniques to improve exercise adherence and thus optimise clinical benefits. A recent systematic review of mobile apps for patients with chronic conditions or multimorbidity has called for further research to develop and evaluate apps that are both high quality and have a high capacity to promote positive behaviour change in patients [26], particularly for people with OA. Significantly, the authors also called for RCTs to test the effectiveness of such apps. Our ongoing trial will address these gaps in the literature and yield important new information about the capacity of theory-informed mobile apps to improve exercise adherence and clinical outcomes in people with knee OA. These RCT findings will be relevant not only to people with knee OA but also to healthcare providers who prescribe or advocate exercise participation to their patients. As the ‘My Exercise Messages’ app is free of charge to consumers in the Apple App Store and Google Play, findings from this study can be easily implemented into practice.

## Data Availability

Datasets analysed for this RCT will be available from the corresponding author upon reasonable request after publication of the trial findings.

## Abbreviations

EARS: Exercise Adherence Rating Scale
HREC: Human Research Ethics Committee
KOOS: Knee Injury and Osteoarthritis Outcome Score
MARS: Mobile Application Rating Scale
NRS: Numerical rating scale
OA: Osteoarthritis
OARSI: Osteoarthritis Research Society International
PASE: Physical Activity Scale for the Elderly
RCT: Randomised controlled trial
SD: Standard deviation
SMS: Short message service
SPIRIT: Standard Protocol Items: Recommendations for Intervention Trials
WOMAC: Western Ontario and McMaster Universities
